# Oxytocin, the Love Hormone, in Stem Cell Differentiation

**DOI:** 10.3390/cimb46110713

**Published:** 2024-10-25

**Authors:** Luca Pampanella, Giovannamaria Petrocelli, Federica Forcellini, Sara Cruciani, Carlo Ventura, Provvidenza Maria Abruzzo, Federica Facchin, Silvia Canaider

**Affiliations:** 1 Department of Medical and Surgical Sciences (DIMEC), University of Bologna, Via Massarenti 9, 40138 Bologna, Italy; luca.pampanella2@unibo.it (L.P.); giovannam.petrocell2@unibo.it (G.P.); federica.forcellini2@studio.unibo.it (F.F.); carlo.ventura@unibo.it (C.V.); silvia.canaider@unibo.it (S.C.); 2 Department of Biomedical Sciences, University of Sassari, Viale San Pietro 43/B, 07100 Sassari, Italy; scruciani@uniss.it; 3 National Laboratory of Molecular Biology and Stem Cell Bioengineering, National Institute of Biostructures and Biosystems (NIBB), Via di Corticella 183, 40129 Bologna, Italy; 4 IRCCS Azienda Ospedaliero-Universitaria di Bologna, Via Massarenti 9, 40138 Bologna, Italy

**Keywords:** oxytocin, oxytocin receptor, stem cells, stem cell differentiation, cardiogenesis, myogenesis, adipogenesis, osteogenesis, chondrogenesis, odontogenesis

## Abstract

Oxytocin (OXT) is a neurohypophysial nonapeptide that exerts its effects mainly through the oxytocin receptor (OXTR). Several studies have pointed out the role of OXT in the modulation of stem cell (SC) fate and properties. SCs are undifferentiated cells characterized by a remarkable ability to self-renew and differentiate into various cell types of the body. In this review, we focused on the role of OXT in SC differentiation. Specifically, we summarize and discuss the scientific research examining the effects of OXT on mesodermal SC-derived lineages, including cardiac, myogenic, adipogenic, osteogenic, and chondrogenic differentiation. The available studies related to the effects of OXT on SC differentiation provide little insights about the molecular mechanism mediated by the OXT–OXTR pathway. Further research is needed to fully elucidate these pathways to effectively modulate SC differentiation and develop potential therapeutic applications in regenerative medicine.

## 1. Introduction

**Figure 1:**
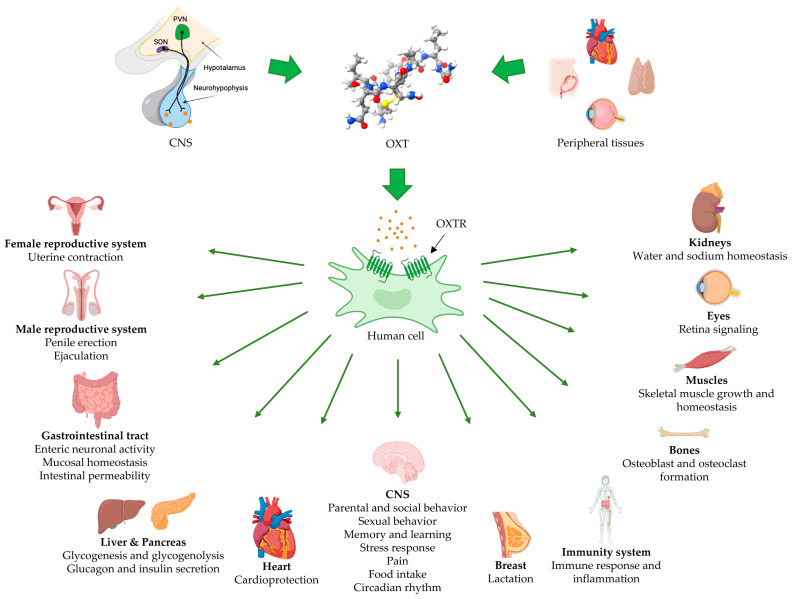
Oxytocin release and its main functions in human tissues and organs. Its 3D molecular structure (National Institute of Health, NIH) is shown as ball-and-stick model using the Jmol variant of Corey–Pauling–Koltun (CPK) coloring: gray = carbon; white = hydrogen; red = oxygen; yellow = sulfur; blue = nitrogen. CNS, central nervous system; OXTR, oxytocin receptor; OXT, oxytocin; PVN, paraventricular nucleus; SON, supraoptic nucleus. Individual images were obtained from BioRender (https://www.biorender.com/, accessed on 23 September 2024).

Oxytocin (OXT) was the first peptide hormone to be structurally determined and chemically synthesized in a biologically active form [1]. OXT is a nonapeptide containing an internal disulfide bond between its Cys(1) and Cys(6) residues. It is mainly synthesized in the magnocellular neurons of the hypothalamic paraventricular and supraoptic nuclei [2], but it is also produced locally in peripheral tissues where it exerts paracrine and autocrine functions (Figure 1) [3]. In response to specific stimuli, OXT is released into the systemic circulation from the posterior pituitary gland and exerts its function by binding to the oxytocin receptor (OXTR) [4]. OXTR belongs to the heptahelical G protein-coupled receptor family, and it is expressed in many tissues like the myometrium, mammary gland, thymus, heart, ovary, kidney, and brain [5]. Upon binding to its receptor, OXT triggers signal transduction, mainly involving the phosphoinositide pathway; this activation increases the levels of intracellular Ca^2+^, which plays a key role in several intracellular functions, such as inducing muscle contractions [6]. As a matter of fact, OXT was first identified for its function in stimulating uterine smooth muscle contractions [7]. During childbirth, the mechanical stimulation from the stretching of the cervix and uterus triggers the release of OXT, which induces uterine contractions, thereby facilitating labor [8]. Beyond its role during parturition, multiple functions of OXT have been described in human physiology (Figure 1) [9,10]. For instance, OXT plays a key role in lactation [4]: breastfeeding activates sensory neurons in the nipple, which send signals via the spinal cord to the hypothalamus. This, in turn, leads to the release of OXT, causing milk ejection [4]. In addition, OXT is often referred to as the “love hormone” due to its association with reproduction, birth and maternal behavior, social bonding, sexual behavior, and emotional well-being [11]. Psychosocial stimuli, such as emotional and social interactions, can also stimulate OXT release and modulate social responses [12]. Furthermore, OXT is involved in other central and peripheral physiological processes [9], including inflammation and immune system regulation [13,14], energy metabolism [15], stress responses [16], pain modulation [4], male reproductive system regulation [17], water homeostasis regulation in the kidney [18], cardioprotection and cardiomyogenesis [19], as well as thermoregulation and body composition regulation [20,21,22,23]. Finally, its other less studied functions are related to other organs such as the pancreas, liver, eye, skin, bone, and skeletal musculature [3,17]. Given the numerous functions of OXT, it is unsurprising that altered levels of OXT have been associated with several diseases and mental health disorders [9]. Low levels of OXT were observed in patients with Autism Spectrum Disorder, a neurodevelopmental condition characterized by deficits in social interaction and communication [24]. Additionally, low levels of OXT were associated with depression in both non-pregnant adults [25] and postpartum women [26], as well as in anxiety disorder [27] and schizophrenia [28]. On the other hand, elevated levels of OXT have been observed in several pathological conditions, including Obsessive Compulsive Disorder (OCD) [29,30], maternal aggression [31], and tumor growth [32]. Numerous experimental and clinical studies suggest that pharmacological modulation of OXT could be a promising therapeutic approach for the treatment of diseases characterized by altered OXT expression [27,28,33]. Among the various functions of OXT, we focused specifically on its relationship with stem cell (SC) differentiation.

SCs are undifferentiated cells characterized by their ability to differentiate into specialized cell types and by their capability to self-renew [34]. Due to these biological properties, SCs play a crucial role in many physiological processes, such as embryonic development, tissue homeostasis, and the repair of damaged tissue [35,36].

Moreover, SCs are used in biological research to study developmental processes and disease pathogenesis, and to aid drug discovery [37].

Furthermore, due to their remarkable self-renewing and differentiation abilities, SCs represent an attractive tool for regenerative medicine, a branch of translational research aimed at restoring damaged cells, tissues, or organs through cell-based therapy or by inducing endogenous repair and regeneration processes [38,39]. Different sources of SCs are available for tissue regeneration. Based on their origin, SCs can be classified into embryonic SCs (ESCs), fetal SCs, adult SCs, and induced pluripotent SCs (iPSCs), each differing in their differentiation potential [34,40]. Due to ethical concerns in ESC usage [34] and to the tumorigenicity risk of the employment of promising iPSCs [41], researchers have increasingly turned their attention to adult SCs, such as mesenchymal SCs (MSCs), which are multipotent and naturally present in adult tissues and organs.

Several preclinical and clinical studies demonstrated that SC therapy, particularly using MSCs, can promote tissue repair in injured organs in vivo. This includes applications in bone repair, cutaneous wound healing, pulpitis, and ischemic cardiac tissue through SC differentiation and the secretion of anti-inflammatory molecules [38,42,43].

Therefore, understanding and controlling SC properties, such as proliferation and differentiation, is one of the main goals of researchers developing cell-based therapies to treat a wide range of diseases. In this context, growing evidence has revealed that OXT is an interesting molecule that is able to modulate SC differentiation. OXT stimulates cardiac differentiation in various SCs [19,44] and promotes chondrogenic commitment [45]. In addition, OXT improves muscle and liver regeneration [46,47] and enhances neurogenesis [48]. It also stimulates osteogenic differentiation by inhibiting adipogenesis in both human adipose-derived mesenchymal SCs (ADSCs) and human bone marrow mesenchymal SCs (BMSCs) [49,50,51,52].

In this review, we thoroughly examine the current literature discussing the modulatory role of OXT on mesodermal SC differentiation capacity, focusing on cardiac, myogenic, adipogenic, osteogenic, chondrogenic, and odontogenic lineages. 

## 2. Search Strategy

**Figure 2:**
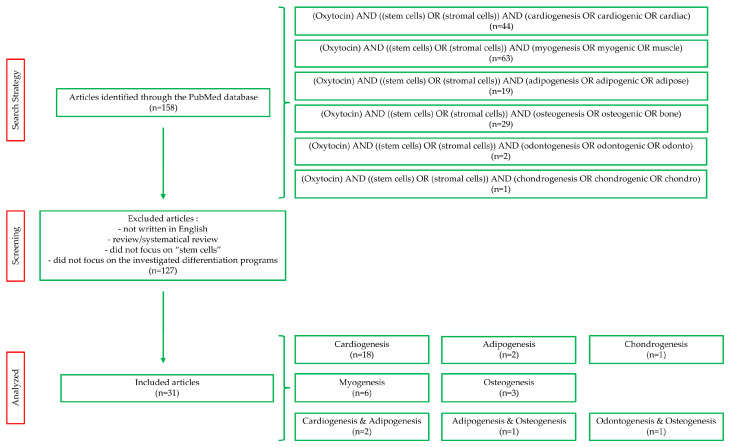
Flow diagram of PubMed search showing number of articles identified (n), according to the declared criteria, and analyzed.

**Figure 3:**
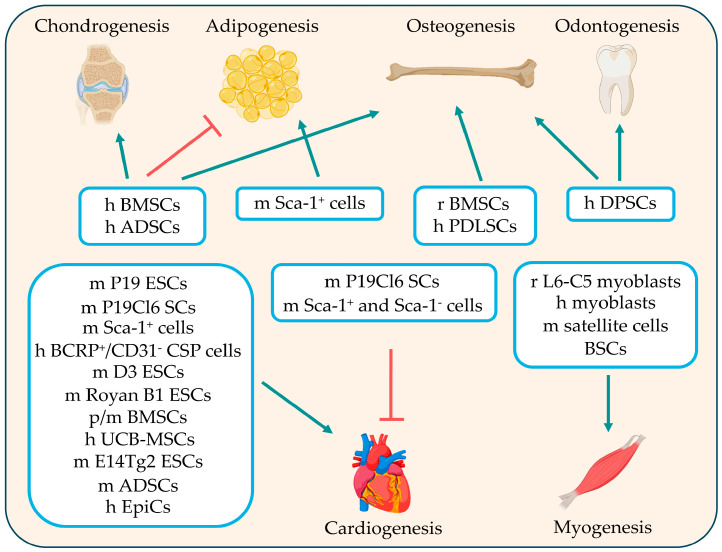
Stem cell types that were treated with oxytocin (OXT) and their differentiation programs. Cells with green arrows are cells prone to differentiation towards the indicated lineages when treated with OXT; cells with red lines are cells that are unable to differentiate into the indicated lineages when treated with OXT. h, human; m, mouse; p, porcine; r, rat. ADSCs, adipose-derived mesenchymal stem cells; BCRP^+^/CD31^−^ CSP cells, breast cancer resistance protein-positive and CD31-negative cardiac side population cells; BMSCs, bone marrow mesenchymal stem cells; BSCs, bovine satellite cells; D3 ESCs, D3 embryonic stem cells; DPSCs, dental pulp stem cells; E14Tg2 ESCs, E14Tg2 embryonic stem cell line; EpiCs, induced pluripotent stem cell-derived epicardial cells; Sca-1^+^/Sca-1^−^ cells, Sca-1-positive/negative cells; L6-C5 myoblasts, mononucleated L6 myoblasts, subclone C5; P19 ESCs, P19 embryonic stem cells; P19Cl6 SCs, P19 clone 6 stem cells; PDLSCs, periodontal ligament-derived stem cells; Royan B1 ESCs, Royan B1 embryonic stem cells; UCB-MSCs, umbilical cord-derived mesenchymal stem cells. Individual images were obtained from BioRender (https://www.biorender.com/, accessed on 23 September 2024).

The search was performed in March 2024 using the publicly available database PubMed (National Centre for Biotechnology Information, NCBI, Bethesda, MD, USA). No filters were applied during this process and the search terms, generated using the Medical Subject Headings (MeSH) database, were combined into the search strings reported in Figure 2. After an initial screening using the exclusion criteria reported in Figure 2, original articles relevant to the aim of our review were included. The contributions of the authors are summarized in the text in chronological order, with some exceptions where appropriate. In addition, we included tables that report experimental details, such as the OXT doses, administration methods, and specific results obtained from the studies (see in the next paragraphs). Furthermore, for easier comprehension, Figure 3 summarizes the cell types in which OXT has been shown to positively or negatively modulate specific differentiation processes.

## 3. Oxytocin in Stem Cell Cardiac Differentiation

In 2022, the first study investigating the potential role of OXT in the differentiation process was published [53]. The elevated OXT levels observed in the fetal and newborn mouse heart, when cardiomyocytes show intense hyperplasia, led the authors to hypothesize a crucial role of this hormone in cardiac differentiation. To test this hypothesis, they used P19 mouse ESCs (P19 ESCs) and observed that OXT stimulated the cells to start beating by day 8 of the differentiation protocol, earlier than the cells treated with dimethyl sulfoxide (DMSO), which is known to promote differentiation into cardiomyocytes by day 12. The cardiomyocyte phenotype was further confirmed by the increase in the expression of *atrial natriuretic peptide* (*ANP*), *sarcomeric myosin heavy chain* (*MHC*), and *dihydropyridine receptor alpha 1* (*DHPR-α1*), as well as by the presence of abundant mitochondria. Notably, OXT-treated P19 ESCs showed an increase in the expression of OXTR, while an OXT antagonist abrogated cardiomyocyte formation, suggesting a key role of OXT in cardiac differentiation [53]. This role was further supported by the presence of high levels of OXT and OXTR proteins in the developing rat hearts at day 21 of gestation and postnatal days 1–4, when cardiomyocytes exhibit intense hyperplasia [54].

We noticed that following this study [53], many researchers used OXT and its differentiation capacity to validate the stemness properties of specific cells employed in their studies. Nonetheless, since these cells had already been confirmed as SCs, the results reported in these scientific articles were included in this review as further evidence of the ability of OXT to induce cardiac differentiation. For example, in a study investigating the stemness of Sca-1-positive (Sca-1^+^) cells isolated from adult mouse hearts, it was reported that OXT induced Sca-1^+^ cells to differentiate into beating cardiomyocytes expressing cardiac gene and protein markers [55]. On the contrary, in another study, it was observed that Sca-1^+^ and Sca-1-negative (Sca-1^−^) cells isolated from adult mouse skeletal muscle and exposed to OXT did not differentiate into cardiomyocytes [56]. OXT led to a reduction in the expression of two cardiac markers, *NK2 homeobox 5* (*Nkx2.5*) and *GATA-binding protein 4* (*GATA4*); this promoted the differentiation of Sca-1^+^ and Sca-1^−^ cells into adipocytes and epithelial cells, respectively [56].

Another research group also performed different experiments to demonstrate the SC properties of the breast cancer resistance protein-positive and CD31-negative (BCRP^+^/CD31^−^) cardiac side population (CSP) cells obtained from neonatal rat hearts [57]. Among them, the authors treated these cells with OXT and found that it induced the differentiation of BCRP^+^/CD31^−^ CSP cells into beating cardiomyocytes after 3 weeks of treatment. These findings were further confirmed by the increased expression of both cardiac markers, such as *Nkx2.5*, *GATA4*, *myocyte-enhancer factor 2C* (*MEF-2C*), and *ANP*, and of contractile proteins, including myosin light chain 2v (MLC-2v), cardiac troponin T (cTnT), and sarcomeric α-actinin [57].

In 2013, OXT was used to assess the differentiation potential of human BCRP^+^/CD31^−^ CSP cells, which represent the cardiac resident progenitor cells. However, these cells did not exhibit spontaneous beating even after 21 days of the differentiation protocol, although they expressed cTnT protein and showed an increase in the levels of *α-MHC* mRNA [58], suggesting that they did not achieve full differentiation.

In 2007, the effect of OXT on murine P19 clone 6 (P19Cl6) cells, a subclone of P19 ESCs, was investigated by several authors, yielding contrasting results. In one research study [59], it was demonstrated that following OXT treatment, P19Cl6 SCs showed a beating phenotype and expressed elevated levels of the mRNA of the cardiomyocyte-specific transcription factor *GATA4* as well as MLC-2v protein.

On the other hand, the ability of OXT to induce cardiac differentiation in P19Cl6 SCs was not replicated in a subsequent study [60], although it was confirmed that OXT induces cardiac differentiation in P19 ESCs. In fact, the modulation of the mRNA and protein expression of cardiac markers, such as *myosin light chain 2a* (*MLC-2a*), *α-MHC*, *β-MHC*, and cardiac troponin I (cTnI), were observed [60]. The cardiac differentiation of OXT-treated P19 ESCs was further validated in 2009 [61]. To understand the different behaviors of P19 and P19Cl6 SCs during cardiac differentiation, OXTR and its relationship with early cardiac marker genes, particularly *GATA4*, were analyzed [60]. The data revealed that although OXT induced an increase in OXTR levels, it failed to stimulate cardiomyogenic differentiation in mP19Cl6 SCs, probably because it was unable to properly induce the expression of *GATA4*, which is essential for this process [60]. 

Conversely, in another study, it was demonstrated that OXT promoted spontaneous beating in both mouse P19 ESCs and the subclone P19Cl6 [62]; the results indicated that nitric oxide (NO) plays a key role in OXT-mediated cardiogenesis, as evidenced by the abrogation of OXT effects when NO synthase was inhibited [62]. The role of NO in OXT-mediated cardiogenic differentiation was also confirmed in 2011 in porcine BMSCs [63]. The study emphasized the importance of treating cells with OXT at early culture passages, when they express high levels of *OXTR* transcripts, which increased their responsiveness to OXT stimulation and improved their differentiation potential [64]. The effects of OXT on the P19Cl6 SC model were also investigated in another research study [65] where the authors focused on the cardiac differentiation of P19Cl6 SCs cultured as confluent monolayers or in aggregates (embryoid bodies, EBs). Interestingly, they found that OXT induced P19Cl6 SCs to differentiate into cardiomyocytes only when they grew in aggregates, whereas it was ineffective when the cells were maintained in a monolayer culture. This finding suggests that cell interactions within EBs influence the cell fate of OXT-treated P19Cl6 SCs [65].

The role of OXT in promoting cardiac differentiation was further demonstrated in murine Royan B1 ESCs. OXT promoted the early maturation of ESC-derived cardiomyocytes, as evidenced by enhanced chronotropic responses and increased expression of cardiac markers, such as cTnI. However, no changes in the ultrastructural characteristics of the cardiomyocytes at any stage of development were observed [66].

In 2008, researchers started to study the involvement of C-terminally extended forms of OXT in cardiogenesis. These forms are derived from the processing of OXT–neurophysin precursors in the hypothalamus and act as intermediate prohormones [67]. In particular, the OXT forms OXT-Gly-Lys-Arg (OXT-GKR), OXT-Gly-Lys (OXT-GK), and OXT-Gly (OXT-G) were used to treat mouse D3 ESCs. OXT-GKR increased the number of beating cells on days 5 and 12, which exhibited a ventricular cell phenotype [67]. Similar results were obtained in cells overexpressing OXT-GKR (OXT-GKR^+^ cells), which showed increased expression of *GATA4* and *MLC-2v* mRNAs. Subsequently, it was demonstrated that OXT-GKR was the dominant form of OXT in newborn rat hearts and it had accumulated concomitantly with OXTR expression in mouse embryos at day 15 [68]. Moreover, OXT-GKR induced contracting cell colonies and more efficiently promoted the expression of ventricular cardiomyocyte markers than OXT and it also reduced the expression of skeletal muscle markers, such as *MEF-2C*, *myogenin *(*MyoG*), and *myogenic differentiation 1* (*MyoD*) mRNAs, in P19 ESCs. These findings led to the hypothesis that the C-terminally extended OXT molecules promote cardiomyocyte differentiation and contribute to heart growth during fetal life [68].

Therefore, in 2014, the same research group treated BCRP^+^/CD31^−^ CSP cells with OXT-GKR [69]. OXT-GKR enhanced cell viability, increased both the formation and size of cell aggregates, induced synchronized contraction of cells, and stimulated the expression of cardiomyocyte markers. Moreover, OXT-GKR induced endothelial differentiation by promoting the formation of a network of tubular cells, the expression of von Willebrand factor (vWF), and the creation of Weibel–Palade bodies, the storage granules in endothelial cells [69].

In a study published in 2010, a pro-migratory effect of OXT on umbilical cord blood-derived MSCs (UCB-MSCs) injected into the myocardium of infarcted rats was reported [70]. In 2012, the same research group evaluated the effect of OXT on UCB-MSCs in vitro [71], demonstrating that, when exposed to OXT, these cells differentiated into cardiomyocytes and expressed cardiac gene and protein markers. Moreover, UCB-MSCs co-cultured with neonatal rat cardiomyocytes subjected to a hypoxia/reoxygenation insult (HR-CMs) showed enhanced expression of cardiac proteins such as connexin 43 (CX43), cTnI, and α-sarcomeric actin (α-SA), indicating that direct contact between UCB-MSCs and HR-CMs may reinforce the effects of OXT on cardiac differentiation [71]. Similar effects were observed in mouse ADSCs, which differentiated into cardiomyocytes when treated with OXT, either alone or in combination with relaxin. Notably, the OXT/relaxin combination enhanced the effects of OXT [72]. The cardiogenic property of OXT was further demonstrated in a murine ESC line (E14Tg2) [73]. OXT reduced the expression of the SC marker *POU domain*, *class 5*, *transcription factor 1* (*OCT4*), while it increased the levels of the mesoderm marker *mesoderm posterior 1* (*Mesp1*) and of cardiogenic markers during the differentiation process. Interestingly, an upregulation of the expression of the *fibroblast growth factor 1* (*FGF1*) gene was observed, particularly in the *FGF1B* transcript levels. FGF1 is expressed in the human fetal heart and plays a role in enhancing cardiac regeneration. Blocking FGF1 actions or its interaction with its receptor, fibroblast growth factor receptor (FGFR), led to a reduction in the efficiency of beating cell formation and in the mRNA levels of cardiomyocyte markers. Moreover, Lin and colleagues observed that inhibiting the FGF1-FGFR downstream effectors AKT serine/threonine kinase (AKT) or protein kinase C (PKC) (particularly PKC ε) further impaired cardiac differentiation, demonstrating that FGF1 could regulate cardiogenesis through PKC signaling [73].

Recently, in a study published in 2022 [74], it was demonstrated that OXT stimulated epicardial cell proliferation, induced epithelial-to-mesenchymal transition (EMT), and increased the transcriptional activity in human induced pluripotent stem cell-derived epicardial cells (hEpiCs). These findings suggested that OXT induces the activation of epicardial cells to a progenitor-like state (hEpiPCs); these multipotent cells play a crucial role in cardiac regeneration after injury, as they can differentiate into various cardiac lineages [74]. The role of OXT in enhancing epicardial cell function was confirmed in a zebrafish model, where OXT levels increased following cardiac cryoinjury. OXT contributed, through the involvement of the transforming growth factor beta (TGF-β) pathway, to epicardial activation and heart regeneration. Furthermore, the authors demonstrated that OXT signaling plays a crucial role in the development of the epicardium in zebrafish embryos [74].

Overall, the studies mentioned above highlight the role of OXT as a key inducer of cardiogenic differentiation in various SC types (Figure 3 and Table 1).

## 4. Oxytocin in Stem Cell Myogenic Differentiation

It is known that OXT exerts anabolic effects on muscle tissue [20,75]. The first study to assess the role of the neurohypophysial nonapeptide arginine8-vasopressin (AVP) and its analogue OXT in myogenic differentiation was conducted in 1995 [76]. In this study, rat mononucleated L6 myoblast cells, subclone C5 (L6-C5), stimulated with OXT acquired multinucleated myotube phenotypic characteristics; the resulting myotubes were larger than those formed in the absence of OXT or AVP and had central nuclei. In addition, the myogenic differentiation capacity of OXT was confirmed at the molecular level by the increased expression of myosin protein [76].

The contribution of the OXT–OXTR pathway to myogenic differentiation was further demonstrated in myoblasts derived from human satellite cells [77]. Myoblasts treated with OXT, AVP, or [Thr(4)Gly(7)]OXT had activated OXTR signaling and showed an increase in the number of fused myoblasts and in the formation of cultured myotubes; this evidence supports the hypothesis that OXT acts in a paracrine/autocrine fashion to stimulate human myoblast fusion [77]. In 2014, the role of OXT in mice skeletal muscles and in their satellite cells was investigated [46]. The findings revealed a decrease in plasma OXT levels in old mice, while OXTR levels in skeletal muscles remained similar between young and old mice. However, a reduction in OXTR expression was observed in old satellite cells. OXT administration improved the capacity of old mice to form new muscle fibers, restoring it to levels comparable to those of young mice, suggesting that OXT administration ameliorated muscle healing in old mice. Moreover, the OXT treatment, by activating the mitogen-activated protein kinase (MAPK)/extracellular signal-regulated kinase (ERK) pathway, increased the proliferation ability of satellite cells in young mice and restored both the proliferation and myogenic differentiation abilities of satellite cells in old mice, as evidenced by the increase in the myogenic fusion index [46].

On the contrary, another study demonstrated that OXT did not influence myogenic fate [78]: murine C2C12 myoblasts were exposed to a chronic treatment with OXT, either alone or in combination with 17β-estradiol (E2), for 7 days during myocyte differentiation [78]. Neither molecule alone or in combination influenced the myotube fusion index, as well as the expression of the *myogenic regulatory factor *(*MRF*) or *MHC* genes in differentiated cells [78]. In another study, while the researchers were studying the effect of 5-azacytidine on human BMSCs cultured on polycaprolactone electrospun fibers for muscle regeneration, they observed that OXT did not affect myogenesis differentiation except when the cells were cultured in the presence of the scaffold, where a positive effect of OXT was observed [79].

In another study [80], the effects of OXT and other steroid hormones on the proliferation and differentiation abilities of bovine satellite cells (BSCs) were compared. OXT was found to increase the fusion index, reduce the number of apoptotic nuclei, enhance BSC migration ability, and upregulate the expression of both *MyoD* and *MyoG* mRNAs. Similar effects were observed in the steroid hormone-treated cells, which increased *OXT* mRNA levels, leading to the hypothesis of a key role of OXT in myogenesis [80]. The results described above are summarized in Figure 3 and Table 2.

## 5. Oxytocin in Stem Cell Adipogenic, Osteogenic, and Odontogenic Differentiation

Osteoblasts and adipocytes originate from the same mesenchymal precursor cells, and an inverse relationship exists between these two lineages. In osteoporosis, bone loss is associated with an increase in bone marrow adipose tissue resulting from the production of adipocytes at the expense of osteoblasts [50]. Thus, identifying signaling pathways that promote MSC osteogenesis and reduce adipogenesis is crucial for reinforcing bone regeneration treatments.

The first study to investigate the role of OXT in these two processes [49] demonstrated that OXT promoted osteoblast differentiation and inhibited adipocyte commitment in both human ADSCs and BMSCs. The authors also speculated that ERK activation might be involved in the OXT-driven differentiation process, while ruling out the involvement of the Ras homolog family member A (RhoA) pathway. Consistent with these findings, ovariectomized (OVX) mice and rats showed a significant decrease in OXT levels, an osteoporotic phenotype, as well as an increase in bone marrow adiposity and an upregulation in the expression of *fatty acid-binding protein 4* (*FABP4*) mRNA, a marker of adipogenesis; these effects were reverted by subcutaneous OXT injections [49]. In another study [81], OVX rats received two implants at the distal femoral metaphysis; the subcutaneous injection of OXT administered after surgery resulting in an enhanced relative bone volume around the implant, an improved percent implant osseointegration, and an increased maximum push-out force and bone mass. These findings indicated that OXT promoted peri-implant bone healing and counteracted the negative effects of osteoporosis.

Consistent with the data obtained in mice, elevated levels of osteoporosis accompanied by increased adipose tissue were observed in rabbits treated with glucocorticoids [82]. OXT treatment reversed the glucocorticoid-induced marrow adiposity and prevented the osteoporosis induced by glucocorticoids.

The inverse relationship between the two lineages was also recently observed in human ADSCs [83]. OXT clearly promoted osteogenic differentiation and either had no effect or sometimes reduced adipogenic differentiation in ADSCs. Moreover, an increase in the expression of the autophagy marker genes *Beclin 1* (*BECN1*) and *Microtubule-Associated Protein 1 Light Chain 3 alpha* (*MAP1LC3A*) was observed at the onset of the osteogenesis, suggesting a role of autophagy in OXT-induced osteogenesis.

Alongside the studies evaluating the effects of OXT in both adipogenesis and osteogenesis, several studies have focused on the role of OXT in only one of these two processes. Regarding adipogenesis, it was observed that Sca-1^+^ cells, previously investigated for their cardiogenic differentiation potential, were able to differentiate into adipocytes when treated with OXT [56].

On the contrary, the data reported in another study showed that OXT did not affect the adipogenic differentiation of mP19 ESCs [61].

Further insights into OXT’s involvement in adipogenesis were further provided by a study focused on the evaluation of *OXTR* mRNA expression during the adipocyte differentiation process [84]. The authors found that *OXTR* mRNA levels were higher in adipocytes derived from mouse adipose tissues compared to vascular stromal cells, and they increased during 3T3-L1 adipocyte differentiation [84].

In addition, it was shown that *OXTR* mRNA expression was higher in older mice and in mice fed with a high-fat diet; moreover, OXT induced lipolysis in 3T3-L1 adipocytes, suggesting a role of OXTR in the regulation of both adipocyte differentiation and fat accumulation [84]. Notably, the effects of OXT on fat and metabolism have also been reported in in vivo models [85,86].

In a study investigating the role of OXT in osteogenesis, it was demonstrated that OXT promoted osteogenic differentiation in BMSCs derived from both cyclic adult (12 months old) and acyclic aging (24 months old) female Wistar rats cultured in osteogenic medium. OXT treatment led to an increase in the expression of both *OXT* and *OXTR*, and anticipated mineralization, and enhanced the gene expression of *bone morphogenetic protein 2* (*BMP2*), *bone sialoprotein* (*BSP*), *osteopontin* (*OPN*), and *osteocalcin* (*OCN*) in both rat populations [87]. Consistent with these findings, OXT also promoted osteogenic differentiation in human periodontal ligament SCs (PDLSCs) [51]. PDLSCs, which express OXTR, increased their migration and proliferation ability upon OXT treatment; in addition, OXT enhanced mineralized nodule formation and calcium deposition and significantly upregulated the expression of osteogenesis-related markers, such as *alkaline phosphatase* (*ALP*), *collagen I* (*Col I*), *runt-related transcription factor 2* (*RUNX2*), *OPN*, and *OCN*. Finally, the authors suggested that OXT exerts its function through the phosphorylation of ERK and AKT proteins [51].

Another study partially challenges the previously reported findings [88]: here, human dental pulp-derived stromal cells (DPSCs) were shown to express OXTR [89]. Upon blocking OXTR using specific antagonists or siRNA, the authors observed an increase in calcium deposition and in the expression of markers of osteogenic (*BMP2*, *OPN*, *OCN*, and *RUNX2*) and odontogenic processes (*dentin matrix acidic phosphoprotein 1*, *DMP1*, and *dentin sialophosphoprotein*, *DSPP*), depending on whether OXT was used together with the osteogenic induction medium or in the basal culture medium [88]. Surprisingly, treatment of the DPSCs with OXT still resulted in an increase in osteogenic differentiation, albeit to a lesser extent.

Moreover, it was found that OXTR is involved in extracellular matrix (ECM) remodeling through modulating the expression of genes related to ECM homeostasis, probably through the Yes-associated protein (YAP) signaling pathway [88]. The results on adipogenesis, osteogenesis, and odontogenesis in cells treated with OXT are summarized in Figure 3 and Table 3.

## 6. Oxytocin in Stem Cell Chondrogenic Differentiation

The relationship between OXT and chondrogenesis was investigated in a recent study aimed at understanding whether OXT plays a role in osteoarthritis (OA) [45]. 

Considering the previously reported findings that demonstrated OXTR expression in chondrocytes and its reduction in patients with OA, along with evidence that OXT treatment restored levels of collagen type II (Col II), which were diminished by OA or specific treatments [87,90], a comprehensive research study, using human ADSCs and BMSCs, was conducted to demonstrate the positive effects of OXT treatment on chondrogenesis [45]. These cells are known to express OXTR and to undergo osteogenic differentiation at the expense of adipogenic differentiation when treated with OXT. When exposed to OXT in the presence of chondrogenic medium, these cells increased the glycosaminoglycan content in the extracellular environment, and increased the expression of *aggrecan* (*ACAN*), *cartilage oligomeric matrix protein* (*COMP*), *SRY-related HMG-box gene 9* (*Sox9*), and *collagen type X *(*Col X*); in contrast, OXT treatment reduced the expression of the fibrous tissue marker *collagen type I αlpha 1 chain* (*COL1A1*) [45]. Also, in a 3D cell pellet culture model used to mimic the in vivo cellular condensation process, differentiated human ADSCs expressed chondrogenic markers. Cells cultured with OXT expressed Sox9 and Col II proteins, indicating the formation of a dense filamentous matrix network surrounding the cells [45]. These findings suggested a role of OXT in chondrogenic differentiation. The results on chondrogenesis in cells treated with OXT are summarized in Figure 3 and Table 3.

## 7. Conclusions

In this review, we highlighted the role of OXT in SC differentiation. OXT influences SC commitment toward various mesodermal lineages, such as cardiac, adipogenic, and osteogenic lineages. The activation of specific differentiation processes mediated by OXT has not yet been fully elucidated. OXT mainly exerts its effects through OXTR, which induces several cellular responses in a cell type-dependent manner [91]. Among these, OXTR stimulates the activation of phospholipase C (PLC), which in turn increases intracellular Ca^2+^ mobilization [19]. Ca^2+^ acts as second messenger and is involved in a wide range of processes relevant for the maintenance of SC properties and differentiation [92].

For instance, Ca^2+^ regulates adipocyte differentiation and has different effects at early or late stages of the process [93], and plays a key role in cardiogenic and osteogenic differentiation [94,95]. It was postulated that Ca^2+^ can regulate SC differentiation by acting on two important processes: epigenetic processes and metabolism [90]. Both processes are controlled in a highly specific manner across various SC types and are essential for maintaining SC identity and facilitating differentiation [92]. The studies available in the literature demonstrating the effects of OXT in SC commitment provide limited insight about the molecular mechanism mediated by the OXT–OXTR pathway. Thus, deeply understanding the molecular pathways activated by the OXT–OXTR pathway in the differentiation of specific SCs could be valuable for the modulation of this process in SCs and for the development of potential therapeutic applications in regenerative medicine.

Unraveling these mechanisms is not only essential for advancing regenerative therapies, but also for understanding how OXT might influence SC differentiation during the development of organs, such as the heart. This knowledge could open new perspectives for managing neonatal complications and enable early interventions in cardiovascular diseases.

## Figures and Tables

**Table 1:** Effects of oxytocin on cardiogenic differentiation.

Oxytocin Formulation/Dose	Species and Cell/Animal Model	Treatment	Cellular Mechanism *	Effect(s) *	Ref.
OXT: 100 nM	m P19 ESCs	4 days, then cells were cultured until day 14 without OXT	↑ spontaneous beating	↑ cardiogenesis	[53]
			↑ ANP mRNA		
			↑ mitochondria number		
			↑ sarcomeric MHC and DHPR-α1 mRNAs		
			↑ OXTR mRNA and protein		
			OXT antagonist (vasoticin) inhibited OXT effects		
OXT: 100 nM	m Sca-1^+^ cells	72 h	↑ spontaneous beating	↑ cardiogenesis	[55]
			↑ sarcomeric structures		
			↑ Nkx2.5, GATA4, MEF-2C, α-MHC, β-MHC, MLC-2a, MLC-2v, and cardiac α-actin mRNAs		
			↑ GATA4, ANP, cTnT, MLC-2v, sarcomeric MHC, CX43, and tropomyosin proteins		
			↑ OXTR mRNA		
			OXT antagonist (vasoticin) inhibited OXT effects		
OXT: 100 nM	m P19 ESCs and m P19Cl6 SCs	EBs treated for 4 days, then cells were cultured until day 14 or 16 without OXT	↑ spontaneous beating	↑ cardiogenesis OXT acts through NO signaling	[62]
			↑ GATA4, ANP, Nkx2.5, MEF-2C, α-MHC, and MyoG mRNAs		
			NOS inhibitor (L-NAME) blocked OXT action		
OXT: 1000 nM	m P19Cl6 SCs	EBs treated for 5 days, then cells were cultured until day 14 without OXT	↑ spontaneous beating	↑ cardiogenesis	[59]
			↑ GATA4 mRNA		
			↑ MLC-2v protein		
			OXTR antagonist (H-9405) reduced MLC-2v protein expression		
OXT: 10 nM	m Royan B1 ESCs	EBs treated for 5 days, then cells were cultured until day 30 without OXT	↑ spontaneous beating	↑ cardiogenesis	[66]
			↑ cTnI protein expression		
			↑ β-MHC, ANP, and MLC-2v mRNAs at early stage of development		
OXT: 100 nM	r BCRP^+^/CD31^−^ CSP cells	72 h	↑ spontaneous beating	↑ cardiogenesis	[57]
			↑ Nkx2.5, GATA4, MEF-2C, β-MHC, and MLC-2c mRNAs		
			↑ GATA4, ANP, cTnT, MLC-2v, and sarcomeric α-actinin proteins		
OXT: 1000, 100 or 10 nM	m P19 ESCs and m P19Cl6 SCs	4 days, then cells were cultured without OXT	↑ spontaneous beating only in P19 cells	↑ cardiogenesis GATA4 upregulation has a key role in cardiogenesis induction	[60]
			↑ MLC-2a, α-MHC, β-MHC, Myf5, MyoD, and MyoG mRNAs (only in P19 cells)		
			↑ cTnI protein (only in P19 cells)		
			↑ GATA4 mRNA (only in P19 cells)		
OXT: 100 nM	m Sca-1^+^ and Sca-1^−^ cells	30 days	↓ Nkx2.5 and GATA4 mRNAs at 4 days in SM^−^ cells	↓ cardiogenesis	[56]
			= Nkx2.5 and GATA4 mRNAs in SM^+^ cells		
			↓ Nkx2.5, GATA4, cMHC, and α-SA proteins in both SM^−^ and SM^+^ cells treated with OXT		
OXT/OXT-GKR/OXT-G/OXT-GK: 1000 nM	m D3 ESCs	EBs treated for 5 days	↑ beating cells at 12 days in OXT- and OXT-GKR-treated cells	↑ cardiogenesis OT-GKR stimulates cells to differentiate toward a ventricular phenotype	[67]
			↑ beating cells at 5 days (only in OXT-GKR-treated cells)		
			OXT antagonist (H-9405) inhibited OXT and OXT-GKR effects		
			↑ GATA4 and MLC-2v mRNAs in OXT-GKR^+^ cells		
			↑ number of ventricular-like cells in OXT-GKR^+^ cells		
			↑ CX43 protein in OXT-GKR^+^ cells		
OXT: 100 nM	m P19 ESCs	4 days	↑ spontaneous beating	↑ cardiogenesis	[61]
			↑ cTpnl and MyoD mRNAs		
OXT: 100 nM	m P19Cl6 SCs	EBs or cells cultured as monolayer treated for 6 or 14 days	↑ spontaneous beating (only in EB cells)	↑ cardiogenesis	[65]
			↑ GATA4, Nkx2.5, α-cardiac actin, β-MHC, Tbx5, and Tbx20 mRNAs		
			↑ β-MHC and cTnI proteins		
OXT/OXT-GKR/OXT-G/OXT-GK: 1000 nM	m P19 ESCs	EBs treated for 5 days	OXT-GKR is a dominant form of OXT in newborn rat hearts	↑ cardiogenesis	[68]
			↑ contracting cells in OXT-GKR-treated cells		
			OXTR silencing inhibited OXT-GKR effects		
			↑ GATA4, MEF-2C, MyoG, and MyoD mRNAs		
			↑ DHPRα1, MLC-2v, and sarcomeric α-actinin proteins		
OXT: 10,000 nM	p BMSCs	1 day, then cells were cultured until day 15 without OXT	↑ eNOS and iNOS mRNAs and proteins	↑ cardiogenesis OXT acts through NO signaling	[63]
			↑ cTnI mRNA		
			NOS inhibitor (L-NAME) reduced cTnI and PLB mRNAs		
			↑ cTnT, cMHC, and cTnI proteins		
			NOS inhibitor (L-NAME) reduced cTnT, cMHC, and cTnI proteins		
			↑ proliferation		
OXT: 100 nM	h UCB-MSCs	7 days	↑ CX43, cTnI, and α-SA proteins at 7 days	↑ cardiogenesis	[71]
			↑ CX43 and cTnT mRNAs and proteins at 4 days		
			↑ eNOS mRNA and protein at 7 days		
OXT: 100 nM	h BCRP^+^/CD31^−^ CSP cells	72 h	No spontaneous beating	↑ cardiogenesis	[58]
			↑ cTnT protein		
			↑ α-MHC mRNA		
OXT-GKR: 100 nM	r BCRP^+^/CD31^−^ CSP cells	EBs treated for 5 days, then cells were cultured until day 18 without OXT	↑ cell viability	↑ cardiogenesis	[69]
			↑ formation and size of EBs		
			↑ synchronized contraction of cells		
			↑ MLC-2v, sarcomeric α-actinin, and cTnT proteins		
			↓ nestin protein		
OXT: 100 nM	m E14Tg2 ESCs	4 days in differentiation medium, then cells were cultured until day 14 without OXT	↓ OCT4 and ↑ Mesp1 mRNAs	↑ cardiogenesis OXT acts through FGF1B and PKC signaling pathways	[73]
			↑ GATA4, cTnT, MLC-2v, and *α*-MHC mRNAs		
			↑ sarcomeric α-actinin, FGF1, cTnT, and *α*-tubulin proteins		
			↑ FGF1B mRNA at the late differentiation stage		
			Blocking FGF1 actions or its receptor impaired cardiac differentiation		
			AKT and PKC inhibitor reduced beating cell cluster formation		
OXT: 10,000 nM	m BMSCs	3 weeks	↑ GATA4, PLB, desmin, and cTnI mRNAs	↑ cardiogenesis	[64]
			↑ cTnT, cTnI, and cMHC proteins		
OXT: 1000, 100 or 10 nM	m ADSCs	4 days, then cells were cultured for up to 3 weeks without OXT	↑ MEF-2c, MLC-2a, MLC-2v, and CX43 mRNA,	↑ cardiogenesis	[72]
			↑ CX43, desmin, and sarcomeric α-actinin proteins		
OXT: 100 nM	In vitro: h EpiCs In vivo: zebrafish embryos and cardiac cryoinjured adult zebrafish	In vitro: 3 days	↑ proliferation in vitro	↑ epicardial cell activation and heart regeneration	[74]
			↑ WT1, TCF21, SNAI1, and NT5E mRNAs in vitro		
			↑ Ki-67, WT1, and TJP1 proteins in vitro		
			Inhibition of OXTR impaired OXT action on Ki-67, WT1, SNAI1, and TJP1 protein expression in vitro		
			↑ activity of TGF-β/BMP pathway biological processes in vitro		
			OXTR inhibition impaired the formation of the epicardium in vivo		
			OXTR inhibition impaired the proliferation and migration of progenitor cells in vivo		
			OXTR inhibition decreased the expression of PCNA and cTnT proteins in vivo		
			OXTR inhibition decreased the mRNA expression of WTB1, TCF21, SNAI1a, and SNAI2 in vivo		

Abbreviations: OXT, oxytocin; OXT-G, OXT-Gly; OXT-GK, OXT-Gly-Lys; OXT-GKR, OXT-Gly-Lys-Arg; OXT-GKR^+^ cells, OXT-GKR-overexpressing cells; h, human; m, mouse; p, porcine; r, rat; ADSCs, adipose-derived mesenchymal stem cells; BCRP^+^/CD31^−^ CSP cells, breast cancer resistance protein-positive and CD31-negative cardiac side population cells; BMSCs, bone marrow mesenchymal stem cells; D3 ESCs, D3 embryonic stem cells; E14Tg2 ESCs, E14Tg2 embryonic stem cells; EpiCs, induced pluripotent stem cell-derived epicardial cells; Sca-1^+^/Sca-1^−^ cells, Sca-1-positive/negative cells; P19 ESCs, P19 embryonic stem cells; P19Cl6 SCs, P19 clone 6 stem cells; Royan B1 ESCs, Royan B1 embryonic stem cells; UCB-MSCs, umbilical cord-derived mesenchymal stem cells; EB, embryonic body; α-MHC, α-myosin heavy chain; α-SA, α-sarcomeric actin; ANP, atrial natriuretic peptide; AKT, serine/threonine kinase; β-MHC, β-myosin heavy chain; BMP, bone morphogenetic protein; cMHC, cardiac myosin heavy chain; cTnI, cardiac troponin I; cTnT, cardiac troponin T; cTpnl, cardiac troponin inhibitor; CX43, connexin 43; DHPR-α1, dihydropyridine receptor-α1; eNOS, endothelial nitric oxide synthase; FGF1, fibroblast growth factor 1; FGF1B, fibroblast growth factor 1B; GATA4, GATA-binding protein 4; H-9405, [β-mercapto-β,β-cyclopentamethylene-propionyl-Tyr(Me)^2^-Ile-Thr-Asn-Cys-Pro-Orn-Tyr-NH_2_]; iNOS, inducible nitric oxide synthase; Ki-67, proliferation marker protein Ki-67; L-NAME, N (G)-nitro-L-arginine methyl ester; MEF-2C, myocyte enhancer factor 2C; Mesp1, mesoderm posterior 1; MLC-2a, myosin light chain 2a; MLC-2c, myosin light chain 2c; MLC-2v, myosin light chain 2v; Myf5, myogenic factor 5; MyoD, myogenic differentiation 1; MyoG, myogenin; Nkx2.5, NK2 homeobox 5; NO, nitric oxide; NOS, nitric oxide synthase; NT5E, 5′-nucleotidase ecto; OCT4, POU domain, class 5, transcription factor 1; OXTR, oxytocin receptor; PKC, protein kinase C; PCNA, proliferating cell nuclear antigen; PLB, phospholamban; sarcomeric MHC, sarcomeric myosin heavy chain; SNAI1, snail family transcriptional repressor 1; SNAI1a, snail family transcriptional repressor 1a; SNAI2, snail family transcriptional repressor 2; Tbx5, T-box transcription factor 5; Tbx20, T-box transcription factor 20; TCF21, transcription factor 21; TGF-β, transforming growth factor beta; TJP1, tight junction protein 1; vasoticin, d(CH_2_)_5_^1^,Tyr(Me)^2^,Thr-4,Orn-8,Tyr-NH_2_^9^; WT1, WT1 transcription factor; WT1B, WT1 transcription factor b. *Arrow and equal symbols indicate the effects of OXT on biological stem cell properties: ↑ (increase), ↓ (reduce), = (no effect).

**Table 2:** Effects of oxytocin on myogenic differentiation.

Oxytocin Formulation/Dose	Species and Cell/Animal Model	Treatment	Cellular Mechanism *	Effect(s) *	Ref.
OXT: 100 nM	r L6-C5 myoblasts	5 or more days	↑ myoblast fusion index	↑ myogenesis	[76]
			↑ myosin protein levels		
OXT: 1000 nM	h myoblasts	20–48 h in differentiation medium	↑ myoblast fusion index	↑ myogenesis	[77]
OXT: 30 nM in vitro; 1 μg/g in vivo	m satellite cells m muscles	In vitro: 24 or 48 h In vivo: daily treatment for 4/6 days before the muscle injury and until animal sacrifice	In vitro: ↑ proliferation of old satellite cells and primary myogenic progenitors	↑ proliferation in vitro ↑ myogenesis in vivo	[46]
			OXT acts via the MAPK/ERK signaling pathway		
			In vivo: ↓ OXT plasma levels with age		
			Similar OXTR protein expression in skeletal muscles between young and old mice		
			↓ OXTR protein expression in satellite cells with age		
			↑ new muscle fiber formation in OXT-treated old mice		
			↑ myogenic cell proliferation in OXT-treated old mice		
			↑ proliferation ability of satellite cells in OXT-treated old and young mice		
			↑ differentiation ability of satellite cells in old mice with OXT administration		
OXT: 10,000 nM	m C2C12 myoblasts	7 days during the myotube differentiation	=myoblast fusion index	=myogenesis	[78]
			=MRF and MHC mRNA levels		
OXT: 10 nM	h BMSCs	28 days	=YIP-1B protein expression	=myogenesis	[79]
OXT: 31.25, 62.5, 125, 250 nM	BSCs	48 or 72 h	↑ myoblast fusion index	↑ myogenesis	[80]
			↓ apoptotic nuclei		
			↑ cell migration		
			↑ MyoD and MyoG mRNAs during BSC proliferation		
			↑ MyoG mRNA during BSC differentiation		

Abbreviations: OXT, oxytocin; h, human; m, mouse; r, rat; BMSCs, bone marrow mesenchymal stem cells; BSCs, bovine satellite cells; L6-C5 myoblasts, mononucleated L6 myoblasts, subclone C5; P19, P19 embryonic stem cell; ERK, extracellular signal-regulated kinase; MAPK, mitogen-activated protein kinase; MHC, myosin heavy chain; MRF, myogenic regulatory factor; MyoD, myogenic differentiation 1; MyoG, myogenin; OXTR, oxytocin receptor; Yip-1B, Yip1-interacting factor homolog B. * Arrow and equal symbols indicate the effects of OXT on biological stem cell properties: ↑ (increase), ↓ (reduce), = (no effect).

**Table 3:** Effects of oxytocin on adipogenic, chondrogenic, osteogenic, and odontogenic differentiation.

Oxytocin Formulation/Dose	Species and Cell/Animal Model	Treatment	Cellular Mechanism *	Effect(s) *	Ref.
OXT: in vitro 30 nM; in vivo 1 mg/kg	in vitro: h ADSCs and h BMSCs in vivo: ovariectomized eight-week-old C57Bl/6J mice and rats	in vitro: in osteogenic or adipogenic differentiation medium; in vivo: daily treatment for 8 weeks	↑ OXTR mRNA during osteogenesis	↓ adipogenesis ↑ osteogenesis	[49]
			↓ OXTR mRNA during adipogenesis		
			↑ mineral deposits		
			↑ ALP activity		
			↑ PDPN mRNA		
			↓ lipid droplet formation		
			↓ GPDH activity		
			↓ FABP4 mRNA		
			↑ ERK1 and ERK2 phosphorylation		
OXT: 100 nM	m Sca-1^+^ and Sca-1^−^ cells	30 days	Sca-1^+^ cells showed scattered cellular aggregates with an adipocytic phenotype	↑ adipogenesis	[56]
OXT: 100 nM	m P19 ESCs	20 days after aggregation in the presence of adipogenic differentiation medium	=lipid droplet formation	=adipogenesis	[61]
			=PPARγ mRNA		
OXT: 1 nM	m 3T3-L1 cells	24 h	↑ OXTR mRNA during adipocyte differentiation;	↑ lipolysis	[84]
			↑ glycerol release		
OXT: 100 nM	r BMSCs	3, 7, and 14 days in the presence of osteogenic medium	↑ calcium deposits at 14 days and at 17 days in cells from 12- and 24-month-old rats, respectively	↑ osteogenesis	[87]
			↑ OXT and OXTR mRNAs in cells from both ages		
			↑ ALP activity in cells from 24 month-old rats		
			↑ BMP2, BSP, OPN, and OCN mRNAs in cells from both ages		
			↑ OSX and COL1A1 mRNAs in cells from 12 month-old rats		
			↓ OSX and COL1A1 in cells from 24 month-old rats		
OXT: 10, 50, 100 nM	h PDLSCs	7, 14, and 21 days in the presence of osteogenic medium	↑ calcium deposits	↑ osteogenesis ERK and AKT signaling pathways are involved in OXT-induced osteogenesis	[51]
			↑ OXTR mRNA		
			↑ ALP, Col I, RUNX2, OPN, and OCN mRNAs		
			↑ ALP, Col I, and RUNX2 proteins		
			↑ ERK and AKT phosphorylation		
			↓ PI3K phosphorylation		
OXT: 30 nM	h BMSCs and h ADSCs	2D or 3D cultures were treated for 21 days in the presence of chondrogenic differentiation medium	↑ glycosaminoglycan content	↑ chondrogenesis	[45]
			↑ ACAN, COMP, SOX9, and Col X mRNAs in 2D culture especially hADSC cultures		
			↓ COL1A1 mRNA in 2D culture of both cell types		
			↑ ACAN, SOX9, and Col X mRNAs in 3D culture of h ADSCs		
			↑ SOX9 and Col II proteins in 3D culture of h ADSCs		
OXT: 300 nM	h DPSCs	2 weeks in osteogenic medium with OXTR inhibitors or OXT	↓ OXTR mRNA during osteogenesis	↑ osteogenesis ↑ odontogenesis	[88]
			↑ calcium deposits by inhibiting OXTR		
			↑ BMP2, OPN, OCN, and RUNX2 mRNAs with OXTR inhibitor (atosiban)		
			↑ DMP1 and DSPP mRNAs with OXTR inhibitor (atosiban)		
			OXT slightly increased calcium deposits		
			OXT slightly increased BMP2, OPN, OCN, RUNX2, and DSPP mRNAs		
			↑ MMP1 mRNA with OXTR inhibitor (atosiban)		
			↓ COL1A1 mRNA with OXTR inhibitor (atosiban)		
			↓ MMP1 mRNA with YAP inhibitor (verteporfin)		
			↑ COL1A1 mRNA with YAP inhibitor (verteporfin)		
			↓ YAP protein in the nucleus of cells with OXTR inhibitor (atosiban)		
			↑ OCN, DSPP, and DMP1 mRNAs by silencing YAP		
			↑ calcium deposits with YAP inhibitor (verteporfin)		
OXT: 100, 500, 1000 nM	h ADSCs	72 h in basal medium treatment lasted for the entire differentiation protocol in the presence of adipogenic or osteogenic medium	↑ OXTR mRNA	↓ adipogenesis ↑ osteogenesis	[83]
			OXT alone did not affect adipogenic and osteogenic differentiation		
			↓ lipid droplet formation in adipogenic medium		
			↓ PPARγ mRNA in adipogenic medium		
			↑ calcium deposits in osteogenic medium		
			↑ OCN mRNA in osteogenic medium		
			RUNX, OPN, fibronectin, and Col I proteins in osteogenic medium		
			↑ BECN1 and MAP1LC3A mRNAs in osteogenic medium		

Abbreviations: OXT, oxytocin; h, human; m, mouse; r, rat; 3T3-L1, 3T3-L1 preadipocytes; ADSCs, adipose-derived mesenchymal stem cells; BMSCs, bone marrow mesenchymal stem cells; DPSCs, dental pulp stem cells; P19 ESCs, P19 embryonic stem cells; PDLSCs, periodontal ligament-derived stem cells; Sca-1^+^/Sca-1^−^ cells, Sca-1-positive/negative cells; ACAN, aggrecan; AKT, serine/threonine kinase; ALP, alkaline phosphatase; BECN1, Beclin 1; OCN, osteocalcin; BMP2, bone morphogenetic protein 2; BSP, bone sialoprotein; ERK1, extracellular signal-regulated kinase 1; COL1A1/Col I, collagen type I alpha 1 chain;/collagen type I; Col II, collagen type II; Col X, collagen type X; COMP, cartilage oligomeric matrix protein; DMP1, dentin matrix acidic phosphoprotein 1; DSPP, dentin sialophosphoprotein; ERK1, extracellular signal-regulated kinase 1; ERK2, extracellular signal-regulated kinase 2; FABP4, fatty acid-binding protein 4; GPDH, glycerol-3-phosphate dehydrogenase; MAP1LC3A, microtubule-associated protein 1 light chain 3 alpha; MAPK, mitogen-activated protein kinase; MMP1, matrix metalloproteinase 1; OPN, osteopontin; OSX, osterix; OXTR, oxytocin receptor; PDPN, podoplanin; PI3K, phosphoinositide 3-kinase; PPARγ, peroxisome proliferator-activated receptor gamma; RUNX2, runt-related transcription factor 2; Sox9, SRY-related HMG-box gene 9; YAP, Yes-associated protein. * Arrow and equal symbols indicate the effects of OXT on biological stem cell properties: ↑ (increase), ↓ (reduce), = (no effect).

## Data Availability

Not applicable.

## References

[1] Du Vigneaud V., Ressler C., Trippett S. (1953). The sequence of amino acids in oxytocin, with a proposal for the structure of oxytocin. J. Biol. Chem..

[2] Meyer-Lindenberg A., Domes G., Kirsch P., Heinrichs M. (2011). Oxytocin and vasopressin in the human brain: Social neuropeptides for translational medicine. Nat. Rev. Neurosci..

[3] Carter C.S., Kenkel W.M., MacLean E.L., Wilson S.R., Perkeyble A.M., Yee J.R., Ferris C.F., Nazarloo H.P., Porges S.W., Davis J.M. (2020). Is oxytocin “Nature’s medicine”?. Pharmacol. Rev..

[4] Augustine R.A., Seymour A.J., Campbell R.E., Grattan D.R., Brown C.H. (2018). Integrative neuro-humoral regulation of oxytocin neuron activity in pregnancy and lactation. J. Neuroendocrinol..

[5] Kimura T., Saji F., Nishimori K., Ogita K., Nakamura H., Koyama M., Murata Y. (2003). Molecular regulation of the oxytocin receptor in peripheral organs. J. Mol. Endocrinol..

[6] Thibonnier M., Berti-Mattera L.N., Dulin N., Conarty D.M., Mattera R. (1998). Signal transduction pathways of the human V1-vascular, V2-renal, V3-pituitary vasopressin and oxytocin receptors. Prog. Brain Res..

[7] Arrowsmith S., Wray S. (2014). Oxytocin: Its mechanism of action and receptor signaling in the myometrium. J. Neuroendocrinol..

[8] Blanks A.M., Thornton S. (2003). The role of oxytocin in parturition. BJOG Int. J. Obstet. Gynaecol..

[9] Perisic M., Woolcock K., Hering A., Mendel H., Muttenthaler M. (2004). Oxytocin and vasopressin signaling in health and disease. Trends Biochem. Sci..

[10] Camerino C. (2023). The Long Way of Oxytocin from the Uterus to the Heart in 70 Years from Its Discovery. Int. J. Mol. Sci..

[11] Carter C.S. (2022). Oxytocin and love: Myths, metaphors and mysteries. Compr. Psychoneuroendocrinology.

[12] Neumann I.D., Landgraf R. (2012). Balance of Brain Oxytocin and Vasopressin: Implications for Anxiety, Depression, and Social Behaviors. Trends Neurosci..

[13] Bordt E.A., Smith C.J., Demarest T.G., Bilbo S.D., Kingsbury M.A. (2019). Mitochondria, Oxytocin, and Vasopressin: Unfolding the Inflammatory Protein Response. Neurotox. Res..

[14] Szeto A., McCabe P.M., Nation D.A., Tabak B.A., Rossetti M.A., McCullough M.E., Schneiderman N., Mendez A.J. (2011). Evaluation of enzyme immunoassay and radioimmunoassay methods for the measurement of plasma oxytocin. Psychosom. Med..

[15] Amri E.Z., Pisani D.F. (2016). Control of bone and fat mass by oxytocin. Horm. Mol. Biol. Clin. Investig..

[16] Russell G., Lightman S. (2019). The human stress response. Nat. Rev. Endocrinol..

[17] Gimpl G., Fahrenholz F. (2001). The oxytocin receptor system: Structure, function, and regulation. Physiol. Rev..

[18] Juul K.V., Bichet D.G., Nielsen S., Nørgaard J.P. (2014). The physiological and pathophysiological functions of renal and extrarenal vasopressin V2 receptors. Am. J. Physiol. Renal Physiol..

[19] Gutkowska J., Jankowski M. (2012). Oxytocin revisited: Its role in cardiovascular regulation. J. Neuroendocrinol..

[20] Camerino C. (2020). The New Frontier in Oxytocin Physiology: The Oxytonic Contraction. Int. J. Mol. Sci..

[21] Camerino C. (2021). Oxytocin Involvement in Body Composition Unveils the True Identity of Oxytocin. Int. J. Mol. Sci..

[22] Conte E., Romano A., De Bellis M., de Ceglia M., Carratù M.R., Gaetani S., Maqoud F., Tricarico D., Camerino C. (2021). Oxtr/TRPV1 expression and acclimation of skeletal muscle to cold-stress in male mice. J. Endocrinol..

[23] Fukushima A., Kataoka N., Nakamura K. (2022). An oxytocinergic neural pathway that stimulates thermogenic and cardiac sympathetic outflow. Cell Rep..

[24] Yamasue H., Domes G. (2018). Oxytocin and Autism Spectrum Disorders. Curr. Top. Behav. Neurosci..

[25] Gordon I., Zagoory-Sharon O., Schneiderman I., Leckman J.F., Weller A., Feldman R. (2008). Oxytocin and cortisol in romantically unattached young adults: Associations with bonding and psychological distress. Psychophysiology.

[26] Jobst A., Krause D., Maiwald C., Härtl K., Myint A.M., Kästner R., Obermeier M., Padberg F., Brücklmeier B., Weidinger E. (2016). Oxytocin course over pregnancy and postpartum period and the association with postpartum depressive symptoms. Arch. Womens Ment. Health.

[27] Yoon S., Kim Y.K. (2020). The Role of the Oxytocin System in Anxiety Disorders. Adv. Exp. Med. Biol..

[28] Goh K.K., Chen C.H., Lane H.Y. (2021). Oxytocin in Schizophrenia: Pathophysiology and Implications for Future Treatment. Int. J. Mol. Sci..

[29] Leckman J.F., Goodman W.K., North W.G., Chappell P.B., Price L.H., Pauls D.L., Anderson G.M., Riddle M.A., McSwiggan-Hardin M., McDougle C.J. (1994). Elevated cerebrospinal fluid levels of oxytocin in obsessive-compulsive disorder: Comparison with tourette’s syndrome and healthy controls. Arch. Gen. Psychiatry.

[30] Marazziti D., Baroni S., Giannaccini G., Catena-Dell’Osso M., Piccinni A., Massimetti G., Dell’Osso L. (2015). Plasma oxytocin levels in untreated adult obsessive-compulsive disorder patients. Neuropsychobiology.

[31] Bosch O.J., Meddle S.L., Beiderbeck D.I., Douglas A.J., Neumann I.D. (2005). Brain oxytocin correlates with maternal aggression: Link to anxiety. J. Neurosci..

[32] Xu H., Fu S., Chen Q., Gu M., Zhou J., Liu C., Chen Y., Wang Z. (2017). The function of oxytocin: A potential biomarker for prostate cancer diagnosis and promoter of prostate cancer. Oncotarget.

[33] Guastella A.J., Boulton K.A., Whitehouse A.J.O., Song Y.J., Thapa R., Gregory S.G., Pokorski I., Granich J., DeMayo M.M., Ambarchi Z. (2023). The effect of oxytocin nasal spray on social interaction in young children with autism: A randomized clinical trial. Mol. Psychiatry.

[34] Zakrzewski W., Dobrzyński M., Szymonowicz M., Rybak Z. (2019). Stem cells: Past, present, and future. Stem Cell Res. Ther..

[35] Wobus A.M., Boheler K.R. (2005). Embryonic stem cells: Prospects for developmental biology and cell therapy. Physiol. Rev..

[36] Mannino G., Russo C., Maugeri G., Musumeci G., Vicario N., Tibullo D., Giuffrida R., Parenti R., Lo Furno D. (2022). Adult stem cell niches for tissue homeostasis. J. Cell Physiol..

[37] Kolios G., Moodley Y. (2013). Introduction to stem cells and regenerative medicine. Respiration.

[38] Suman S., Domingues A., Ratajczak J., Ratajczak M.Z. (2019). Potential Clinical Applications of Stem Cells in Regenerative Medicine. Adv. Exp. Med. Biol..

[39] Amabile G., Meissner A. (2009). Induced pluripotent stem cells: Current progress and potential for regenerative medicine. Trends Mol. Med..

[40] Sarkar A., Saha S., Paul A., Maji A., Roy P., Maity T.K. (2021). Understanding stem cells and its pivotal role in regenerative medicine. Life Sci..

[41] Takahashi K., Yamanaka S. (2006). Induction of pluripotent stem cells from mouse embryonic and adult fibroblast cultures by defined factors. Cell.

[42] Margiana R., Markov A., Zekiy A.O., Hamza M.U., Al-Dabbagh K.A., Al-Zubaidi S.H., Hameed N.M., Ahmad I., Sivaraman R., Kzar H.H. (2022). Clinical application of mesenchymal stem cell in regenerative medicine: A narrative review. Stem Cell Res. Ther..

[43] Petrosyan A., Martins P.N., Solez K., Uygun B.E., Gorantla V.S., Orlando G. (2022). Regenerative medicine applications: An overview of clinical trials. Front. Bioeng. Biotechnol..

[44] Gutkowska J., Jankowski M., Antunes-Rodrigues J. (2014). The role of oxytocin in cardiovascular regulation. Braz. J. Med. Biol. Res..

[45] Roux C.H., Pisani D.F., Gillet P., Fontas E., Yahia H.B., Djedaini M., Ambrosetti D., Michiels J.F., Panaia-Ferrari P., Breuil V. (2020). Oxytocin Controls Chondrogenesis and Correlates with Osteoarthritis. Int. J. Mol. Sci..

[46] Elabd C., Cousin W., Upadhyayula P., Chen R.Y., Chooljian M.S., Li J., Kung S., Jiang K.P., Conboy I.M. (2014). Oxytocin is an age-specific circulating hormone that is necessary for muscle maintenance and regeneration. Nat. Commun..

[47] Luo D., Jin B., Zhai X., Li J., Liu C., Guo W., Li J. (2021). Oxytocin promotes hepatic regeneration in elderly mice. iScience.

[48] Jafarzadeh N., Javeri A., Khaleghi M., Taha M.F. (2014). Oxytocin improves proliferation and neural differentiation of adipose tissue-derived stem cells. Neurosci. Lett..

[49] Elabd C., Basillais A., Beaupied H., Breuil V., Wagner N., Scheideler M., Zaragosi L.E., Massiéra F., Lemichez E., Trajanoski Z. (2008). Oxytocin controls differentiation of human mesenchymal stem cells and reverses osteoporosis. Stem Cells.

[50] Rosen C.J., Bouxsein M.L. (2006). Mechanisms of disease: Is osteoporosis the obesity of bone?. Nat. Clin. Pract. Rheumatol..

[51] Ge B., Liu H., Liang Q., Shang L., Wang T., Ge S. (2019). Oxytocin facilitates the proliferation, migration and osteogenic differentiation of human periodontal stem cells in vitro. Arch. Oral. Biol..

[52] Seo B.M., Miura M., Gronthos S., Bartold P.M., Batouli S., Brahim J., Young M., Robey P.G., Wang C.Y., Shi S. (2004). Investigation of multipotent postnatal stem cells from human periodontal ligament. Lancet.

[53] Paquin J., Danalache B.A., Jankowski M., McCann S.M., Gutkowska J. (2002). Oxytocin induces differentiation of P19 embryonic stem cells to cardiomyocytes. Proc. Natl. Acad. Sci. USA.

[54] Jankowski M., Danalache B., Wang D., Bhat P., Hajjar F., Marcinkiewicz M., Paquin J., McCann S.M., Gutkowska J. (2004). Oxytocin in cardiac ontogeny. Proc. Natl. Acad. Sci. USA.

[55] Matsuura K., Nagai T., Nishigaki N., Oyama T., Nishi J., Wada H., Sano M., Toko H., Akazawa H., Sato T. (2004). Adult cardiac Sca-1-positive cells differentiate into beating cardiomyocytes. J. Biol. Chem..

[56] Abdel-Latif A., Zuba-Surma E.K., Case J., Tiwari S., Hunt G., Ranjan S., Vincent R.J., Srour E.F., Bolli R., Dawn B. (2008). TGF-beta1 enhances cardiomyogenic differentiation of skeletal muscle-derived adult primitive cells. Basic. Res. Cardiol..

[57] Oyama T., Nagai T., Wada H., Naito A.T., Matsuura K., Iwanaga K., Takahashi T., Goto M., Mikami Y., Yasuda N. (2007). Cardiac side population cells have a potential to migrate and differentiate into cardiomyocytes in vitro and in vivo. J. Cell Biol..

[58] Emmert M.Y., Emmert L.S., Martens A., Ismail I., Schmidt-Richter I., Gawol A., Seifert B., Haverich A., Martin U., Gruh I. (2013). Higher frequencies of BCRP+ cardiac resident cells in ischaemic human myocardium. Eur. Heart J..

[59] Gutkowska J., Miszkurka M., Danalache B., Gassanov N., Wang D., Jankowski M. (2007). Functional arginine vasopressin system in early heart maturation. Am. J. Physiol. Heart Circ. Physiol..

[60] Uchida S., Fuke S., Tsukahara T. (2007). Upregulations of Gata4 and oxytocin receptor are important in cardiomyocyte differentiation processes of P19CL6 cells. J. Cell Biochem..

[61] Bouchard F., Paquin J. (2009). Skeletal and cardiac myogenesis accompany adipogenesis in P19 embryonal stem cells. Stem Cells Dev..

[62] Danalache B.A., Paquin J., Donghao W., Grygorczyk R., Moore J.C., Mummery C.L., Gutkowska J., Jankowski M. (2007). Nitric oxide signaling in oxytocin-mediated cardiomyogenesis. Stem Cells.

[63] Ybarra N., del Castillo J.R., Troncy E. (2011). Involvement of the nitric oxide-soluble guanylyl cyclase pathway in the oxytocin-mediated differentiation of porcine bone marrow stem cells into cardiomyocytes. Nitric Oxide.

[64] Ybarra N., Vincent P., Smith L.C., Troncy E. (2015). Oxytocin improves the expression of cardiac specific markers in porcine bone marrow stem cells differentiation. Res. Vet. Sci..

[65] Fathi F., Murasawa S., Hasegawa S., Asahara T., Kermani A.J., Mowla S.J. (2009). Cardiac differentiation of P19CL6 cells by oxytocin. Int. J. Cardiol..

[66] Hatami L., Valojerdi M.R., Mowla S.J. (2007). Effects of oxytocin on cardiomyocyte differentiation from mouse embryonic stem cells. Int. J. Cardiol..

[67] Gassanov N., Devost D., Danalache B., Noiseux N., Jankowski M., Zingg H.H., Gutkowska J. (2008). Functional activity of the carboxyl-terminally extended oxytocin precursor Peptide during cardiac differentiation of embryonic stem cells. Stem Cells.

[68] Danalache B.A., Gutkowska J., Slusarz M.J., Berezowska I., Jankowski M. (2010). Oxytocin-Gly-Lys-Arg: A novel cardiomyogenic peptide. PLoS ONE.

[69] Danalache B.A., Yu C., Gutkowska J., Jankowski M. (2014). Oxytocin-Gly-Lys-Arg stimulates cardiomyogenesis by targeting cardiac side population cells. J. Endocrinol..

[70] Kim Y.S., Kwon J.S., Hong M.H., Kim J., Song C.H., Jeong M.H., Cho J.G., Park J.C., Kang J.C., Ahn Y. (2010). Promigratory activity of oxytocin on umbilical cord blood-derived mesenchymal stem cells. Artif. Organs.

[71] Kim Y.S., Ahn Y., Kwon J.S., Cho Y.K., Jeong M.H., Cho J.G., Park J.C., Kang J.C. (2012). Priming of mesenchymal stem cells with oxytocin enhances the cardiac repair in ischemia/reperfusion injury. Cells Tissues Organs.

[72] Taha M.F., Javeri A., Karimipour M., Yamaghani M.S. (2019). Priming with oxytocin and relaxin improves cardiac differentiation of adipose tissue-derived stem cells. J. Cell Biochem..

[73] Lin H.Y., Lee D.C., Wang H.D., Chi Y.H., Chiu I.M. (2015). Activation of FGF1B Promoter and FGF1 Are Involved in Cardiogenesis Through the Signaling of PKC, but Not MAPK. Stem Cells Dev..

[74] Wasserman A.H., Huang A.R., Lewis-Israeli Y.R., Dooley M.D., Mitchell A.L., Venkatesan M., Aguirre A. (2022). Oxytocin promotes epicardial cell activation and heart regeneration after cardiac injury. Front. Cell Dev. Biol..

[75] Adamo S., Pigna E., Lugarà R., Moresi V., Coletti D., Bouché M. (2019). Skeletal Muscle: A Significant Novel Neurohypophyseal Hormone-Secreting Organ. Front. Physiol..

[76] Nervi C., Benedetti L., Minasi A., Molinaro M., Adamo S. (1995). Arginine-vasopressin induces differentiation of skeletal myogenic cells and up-regulation of myogenin and Myf-5. Cell Growth Differ..

[77] Breton C., Haenggeli C., Barberis C., Heitz F., Bader C.R., Bernheim L., Tribollet E. (2002). Presence of functional oxytocin receptors in cultured human myoblasts. J. Clin. Endocrinol. Metab..

[78] Berio E., Divari S., Starvaggi Cucuzza L., Biolatti B., Cannizzo F.T. (2017). 17β-estradiol upregulates oxytocin and the oxytocin receptor in C2C12 myotubes. PeerJ.

[79] Fasolino I., Guarino V., Cirillo V., Ambrosio L. (2017). 5-Azacytidine-mediated hMSC behavior on electrospun scaffolds for skeletal muscle regeneration. J. Biomed. Mater. Res. A.

[80] Zhang Z., Zhao L.D., Johnson S.E., Rhoads M.L., Jiang H., Rhoads R.P. (2019). Oxytocin is involved in steroid hormone-stimulated bovine satellite cell proliferation and differentiation in vitro. Domest. Anim. Endocrinol..

[81] Wang M., Lan L., Li T., Li J., Li Y. (2016). The effect of oxytocin on osseointegration of titanium implant in ovariectomized rats. Connect. Tissue Res..

[82] Li B., Jiang Y., Sun J., Liang J., Jin Y. (2016). MR spectroscopy for assessing the effects of oxytocin on marrow adipogenesis induced by glucocorticoid in rabbits. Acta Radiol..

[83] Petrocelli G., Abruzzo P.M., Pampanella L., Tassinari R., Marini S., Zamagni E., Ventura C., Facchin F., Canaider S. (2023). Oxytocin Modulates Osteogenic Commitment in Human Adipose-Derived Stem Cells. Int. J. Mol. Sci..

[84] Yi K.J., So K.H., Hata Y., Suzuki Y., Kato D., Watanabe K., Aso H., Kasahara Y., Nishimori K., Chen C. (2015). The regulation of oxytocin receptor gene expression during adipogenesis. J. Neuroendocrinol..

[85] Camerino C. (2009). Low sympathetic tone and obese phenotype in oxytocin-deficient mice. Obesity.

[86] Takayanagi Y., Kasahara Y., Onaka T., Takahashi N., Kawada T., Nishimori K. (2008). Oxytocin receptor-deficient mice developed late-onset obesity. Neuroreport.

[87] Santos L.F., Singulani M.P., Stringhetta-Garcia C.T., Oliveira S.H.P., Chaves-Neto A.H., Dornelles R.C.M. (2018). Oxytocin effects on osteoblastic differentiation of Bone Marrow Mesenchymal Stem Cells from adult and aging female Wistar rats. Exp. Gerontol..

[88] Jung J.W., Park S.Y., Seo E.J., Jang I.H., Park Y., Lee D., Kim D., Kim J.M. (2023). Functional expression of oxytocin receptors in pulp-dentin complex. Biomaterials.

[89] Kumagai T., Shindo S., Takeda K., Shiba H. (2022). Oxytocin suppresses CXCL10 production in TNF-α-stimulated human dental pulp stem cells. Cell Biol. Int..

[90] Wu Y., Wu T., Xu B., Xu X., Chen H., Li X. (2017). Oxytocin prevents cartilage matrix destruction via regulating matrix metalloproteinases. Biochem. Biophys. Res. Commun..

[91] McKay E.C., Counts S.E. (2020). Oxytocin Receptor Signaling in Vascular Function and Stroke. Front. Neurosci..

[92] Snoeck H.W. (2020). Calcium regulation of stem cells. EMBO Rep..

[93] Zhang F., Ye J., Zhu X., Wang L., Gao P., Shu G., Jiang Q., Wang S. (2019). Anti-Obesity Effects of Dietary Calcium: The Evidence and Possible Mechanisms. Int. J. Mol. Sci..

[94] Tyser R.C., Miranda A.M., Chen C.M., Davidson S.M., Srinivas S., Riley P.R. (2016). Calcium handling precedes cardiac differentiation to initiate the first heartbeat. eLife.

[95] Barradas A.M., Fernandes H.A., Groen N., Chai Y.C., Schrooten J., van de Peppel J., van Leeuwen J.P., van Blitterswijk C.A., de Boer J. (2012). A calcium-induced signaling cascade leading to osteogenic differentiation of human bone marrow-derived mesenchymal stromal cells. Biomaterials.

